# Three-stage limb salvage in tibial fracture related infection with composite bone and soft-tissue defect

**DOI:** 10.1007/s00402-021-04299-9

**Published:** 2021-12-22

**Authors:** Pablo S. Corona, Carla Carbonell-Rosell, Matías Vicente, Jordi Serracanta, Kevin Tetsworth, Vaida Glatt

**Affiliations:** 1 Orthopaedic Surgery Department, Vall d’Hebron University Hospital, Universitat Autònoma de Barcelona, Barcelona, Spain; 2 Septic and Reconstructive Surgery Unit (UCSO), Orthopaedic Surgery Department, Vall d’Hebron University Hospital, Barcelona, Spain; 3 Musculoskeletal Tissue Engineering Group, Vall d’Hebron Research Institute, Universitat Autònoma de Barcelona, Barcelona, Spain; 4 Department of Plastic Surgery and Major Burn, Vall d’Hebron University Hospital, Universitat Autònoma de Barcelona, Barcelona, Spain; 5 Department of Orthopaedic Surgery, Royal Brisbane and Women’s Hospital, Brisbane, Australia; 6 Orthopaedic Research Centre of Australia, Brisbane, Australia; 7 Department of Orthopaedic Surgery, University of Texas Health Science Center San Antonio, San Antonio, TX USA

**Keywords:** Bone transport, Infected tibial injury, Limb salvage, Limb-threatening injuries, Distraction osteogenesis, Fasciocutaneous free flap

## Abstract

**Introduction:**

Managing critical-sized tibial defects is one of the most complex challenges orthopedic surgeons face. This is even more problematic in the presence of infection and soft-tissue loss. The purpose of this study is to describe a comprehensive three-stage surgical protocol for the reconstruction of infected tibial injuries with combined bone defects and soft-tissue loss, and report the clinical outcomes.

**Materials and methods:**

A retrospective study at a specialized limb reconstruction center identified all patients with infected tibial injuries with bone and soft-tissue loss from 2010 through 2018. Thirty-one patients were included. All cases were treated using a three-stage protocol: (1) infected limb damage control; (2) soft-tissue coverage with a vascularized or local flap; (3) definitive bone reconstruction using distraction osteogenesis principles with external fixation. Primary outcomes: limb salvage rate and infection eradication. Secondary outcomes: patient functional outcomes and satisfaction.

**Results:**

Patients in this series of chronically infected tibias had been operated upon 3.4 times on average before starting our limb salvage protocol. The mean soft-tissue and bone defect sizes were 124 cm^2^ (6–600) and 5.4 cm (1–23), respectively. A free flap was performed in 67.7% (21/31) of the cases; bone transport was the selected bone-reconstructive option in 51.7% (15/31). Local flap failure rate was 30% (3/10), with 9.5% for free flaps (2/21). Limb salvage rate was 93.5% (29/31), with infection eradicated in all salvaged limbs. ASAMI bone score: 100% good/excellent. Mean VAS score was 1.0, and ASAMI functional score was good/excellent in 86% of cases. Return-to-work rate was 83%; 86% were “very satisfied” with the treatment outcome.

**Conclusion:**

A three-stage surgical approach to treat chronically infected tibial injuries with combined bone and soft-tissue defects yields high rates of infection eradication and successful limb salvage, with favorable functional outcomes and patient satisfaction.

## Introduction

The tibia is frequently subjected to severe trauma, sometimes resulting in combined bone and soft-tissue loss [1]. Despite adequate initial treatment, complications including fracture, non-union and infection are common [2]. Resulting either from the trauma itself, or subsequent surgical debridement, there may be substantial bone loss, and reconstruction of these defects remains one of the most difficult challenges in orthopedic surgery. This complexity is compounded when there is concomitant soft-tissue loss and infection, and amputation is sometimes a reasonable alternative [3].

With such devastating limb-threatening injuries, the fundamental question is, inevitably, the debate between salvage and amputation [3, 4]. These patients need to be treated at a center experienced in bone and joint infection. The interaction between various specialists as part of an orthoplastic treatment concept allows a simultaneous multidisciplinary approach while the patient is located at a single institution. There is some consensus that limb salvage is preferable to primary amputation [4] whenever there is an expectation that it can be achieved in a reasonable time frame with low risk of infection, while maintaining the probability of satisfactory functional outcomes.

To achieve limb salvage, one must eliminate infection and restore both soft-tissue and bone continuity while preserving adequate limb function [5]. Reconstruction can be difficult and complex, but typically requires radical debridement of all necrotic bone and soft tissues; this can lead to larger bone defects and the need for soft-tissue reconstruction. Despite extensive literature devoted to the topic [5, 6], the timing of address to the different components of the problem (infection, soft-tissue reconstruction, and bone defect) remains a matter of debate [7]. Management can either be completed in a single procedure or under a staged protocol; both approaches have their advantages and disadvantages [5–9]. The final outcome is influenced by additional variables, including pre-existing host factors, impaired local vascularity, the mechanism of injury, microbiology, and patient expectations [7].

Based on our experience in managing patients with complex infected tibial injuries with combined bone and soft-tissue defects, we have refined a comprehensive three-stage limb-salvage treatment protocol. The purpose of this study is to describe this protocol, and report on its clinical outcomes. The primary outcome measures were limb-salvage rate, and the rate of successful infection eradication. Secondary outcomes document bone defect reconstruction techniques, soft-tissue reconstruction procedures, objective functional results, and subjective patient-reported satisfaction. The hypothesis is that this protocol is associated with a high rate of limb salvage, a low rate of residual infection, and that it consistently delivers both high levels of patient satisfaction and favorable functional outcomes.

## Methods

Following institutional review board (IRB) approval, this study was conducted using a retrospective analysis design. Our institutional database was reviewed to identify all patients with infected tibial injuries (posttraumatic or postsurgical) with combined bone and soft-tissue defects, treated under this three-stage protocol between January 2010 and December 2018. In all cases, soft-tissue reconstruction was performed with either vascularized free flaps or a local rotational flap; bone reconstruction was performed using external fixation based on distraction osteogenesis (DO) principles.

All patients had confirmed deep infection according to the internationally accepted definition [10], including at least one of the following criteria: (1) sinus tract; (2) bone or implant exposure; (3) positive histology; (4) gross pus or intraoperative abscess; (5) ≥ 2 positive cultures for the same pathogen. Final confirmation of infection eradication was only made if the patient met all the following criteria: no further intervention related to infection; no death related to infection; no need for suppressive antibiotic treatment, and no persistent clinical signs of infection [11].

Exclusion criteria: pediatric patients; first surgical procedure cultures unavailable; infected bone defects without soft-tissue defects, and less than 12 months of follow-up after completing treatment.

Final clinical assessment included the Visual Analogue Scale (VAS), return to work (RTW) status, the Self-Administered Patient Satisfaction Scale (SAPS) [12], and the Association for the Study and Application of the Methods of Ilizarov (ASAMI) classification [13]. Final patient functional outcome was based on the 20-item Lower-Extremity Functional Scale (LEFS), assessing patient ability to perform everyday tasks [14].

Three different external fixators were used: either a monolateral rail (LRS-Advance^®^, Orthofix, Verona, Italy), an Ilizarov-type circular frame (Truelok^®^, Orthofix, Verona, Italy, and ClickIt^®^-CF, Mikai S.p.A, Genova, Italy), or a hexapod frame (Truelok^®^ Hex, Orthofix, Verona, Italy), selected based on specific case characteristics.

## Three-stage surgical treatment protocol (TSP)

**Fig. 1:**
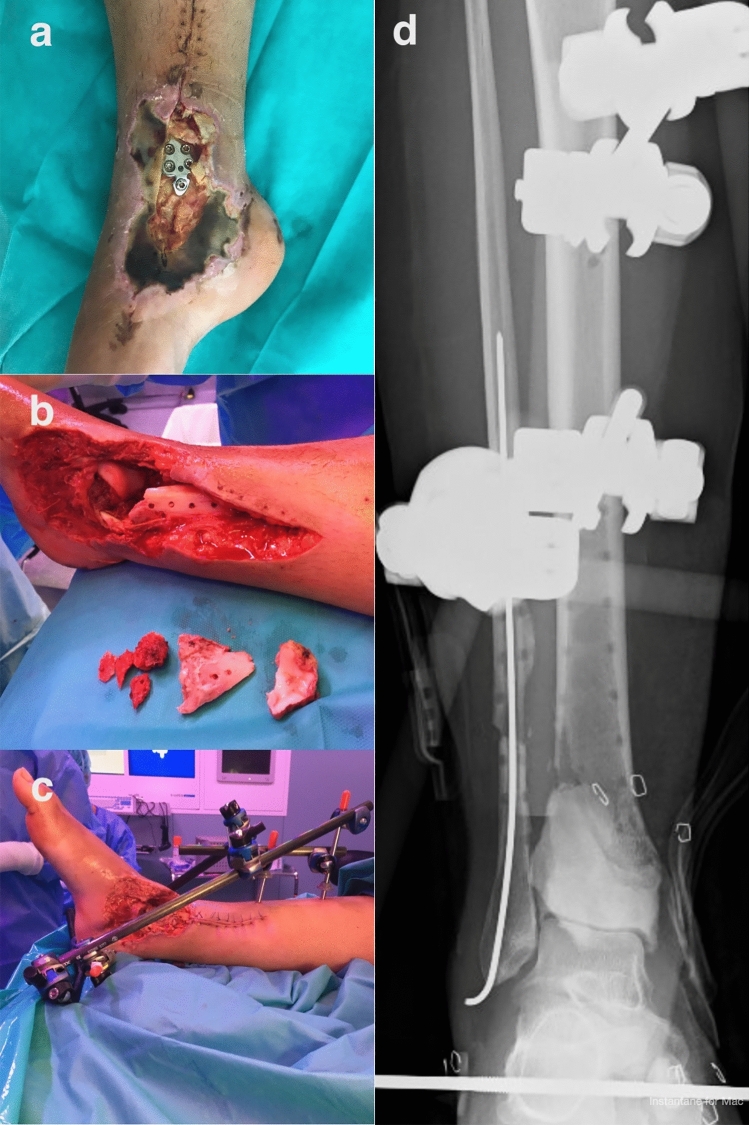
Panel of images demonstrating the 3-stage protocol for management of an infected distal tibial fracture initially stabilized with a plate; **a** preoperative clinical image of a 32-year-old woman with a massive infected soft-tissue defect and a long segment of grossly necrotic bone (with joint involvement); **b** intra-operative image following radical debridement, resulting in a critical segmental bone defect (5 cm) now replaced with antibiotic-laden PMMA; **c** intra-operative image demonstrating a cement spacer together with a temporary (< 10 days) modular external fixator used to obliterate dead space and confer stability; **d** post-operative radiograph after completing the first stage; negative pressure wound therapy (NPWT) device applied to isolate the wound between first and second stages

Under this protocol, surgical management is temporally divided into three discrete treatment phases. The first stage is labeled infected-Limb Damage Control (iLDC), where the goal is infection eradication and extremity resuscitation (Fig. 1). Surgical debridement is the focus, requiring removal of all contaminated hardware and macroscopic devitalized bone and soft tissue [5, 6]. Resection of infected tissue is undertaken from an oncologic perspective to obtain 5 mm margins of healthy tissue (Fig. 1b). To eliminate dead space, a case-specific polymethylmethacrylate (PMMA) spacer is made using antibiotic-loaded acrylic cement (*Vancogenx*^*®*^; Tecres; Verona, Italy), with the addition of extra powdered vancomycin and tobramycin. PMMA is preferred as it effectively obliterates dead space, achieves high concentrations of local antibiotics without risk of systemic toxicity [15], enhances stability, and is easily removed during subsequent stages. An interim modular external fixator (Galaxy™; Orthofix; Verona, Italy or ClickIt^®^-ER, Mikai S.p.A, Genova, Italy) is normally applied (Fig. 1c, d). Temporary wound coverage is provided, generally using negative pressure wound therapy (NPWT). Consistent postoperative antibiotic protocols were administered under the direction of infectious disease consultants as part of our dedicated multidisciplinary unit. In general, antibiotic treatment was selected according to the susceptibility profile of the isolated bacteria. If the pathogen is known prior to first debridement surgery, a targeted prophylaxis after intraoperative sampling is used; the patient starts with directed intravenous antibiotic therapy that can be modified depending on the results of intraoperative cultures. If, after a minimum of 10 days with intravenous treatment, an oral antibiotic with good bioavailability and correct bone penetration is available, antibiotic treatment is switched to oral. In such a pseudo-oncological approach, long-term antibiotic treatment is not usually needed, with an average antibiotic treatment of 4–6 week in total. The reason for staging this phase is to reduce physiological stress for the patient and the very compromised limb, ensure the correct etiological diagnosis of the infection, check the evolution of the soft tissues in order to design the most appropriate type of skin reconstruction, and lower the bacterial load as much as possible prior to the second stage of reconstruction.

The second surgical stage is called the Wound Reconstruction Phase (WRP); it involves formal procedures to achieve durable, pliable, soft-tissue reconstruction. Healthy soft-tissue reconstruction results in a biological chamber that prevents recolonization, acts as antibiotic carrier and promotes bone healing, a fundamental principle of treatment [5, 6]. In all patients a CT-angiography is requested to check the vascular anatomy for a possible microsurgical flap. The patient is evaluated together with an expert in plastic-reconstructive surgery who is part of our dedicated multidisciplinary unit, in an orthoplastic approach concept. Inability to achieve effective coverage is an indication that limb salvage should be abandoned, and amputation recommended. Ideally, this second phase should be performed within 10 days after the first surgery. During this second stage, repeat debridement is performed, the spacer is changed, and deep microbiology samples are again collected. The type of flap is selected based on clinical considerations, anticipating the need to later lift the flap to complete osseous reconstruction.

**Fig. 2:**
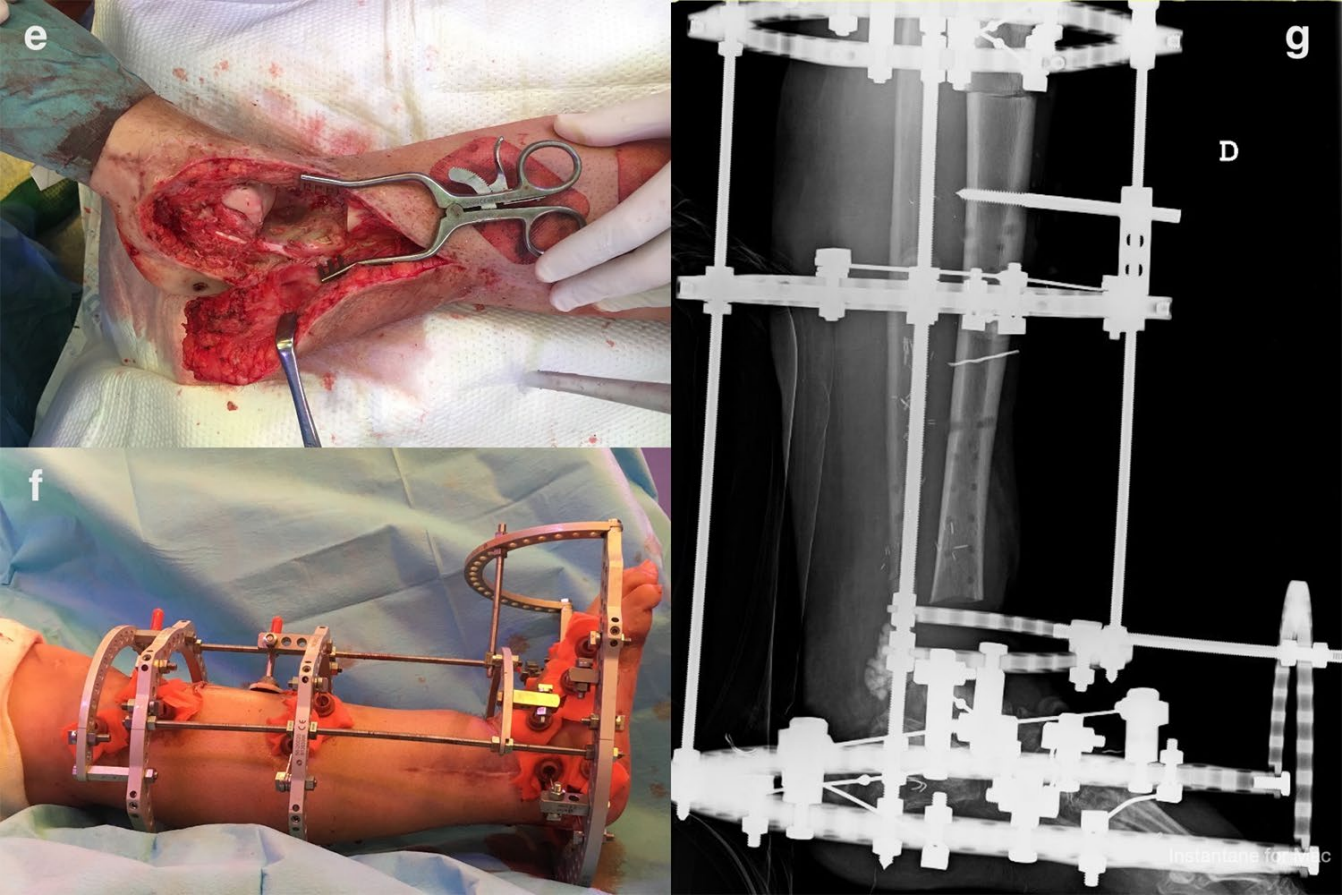
Panel of images demonstrating the 3-Stage Protocol; **e** third-stage procedure, previous (second stage) soft-tissue coverage obtained through a free anterolateral thigh (ALT) flap to reconstruct the massive soft-tissue defect; during the third stage, the flap is lifted and the spacer is extracted (notice the dome of the talus); **f** definitive reconstruction of the segmental bone defect is achieved using a tibiotalar arthrodesis through circular-frame bone transport; **g** postoperative radiograph after the third stage has been completed

**Fig. 3:**
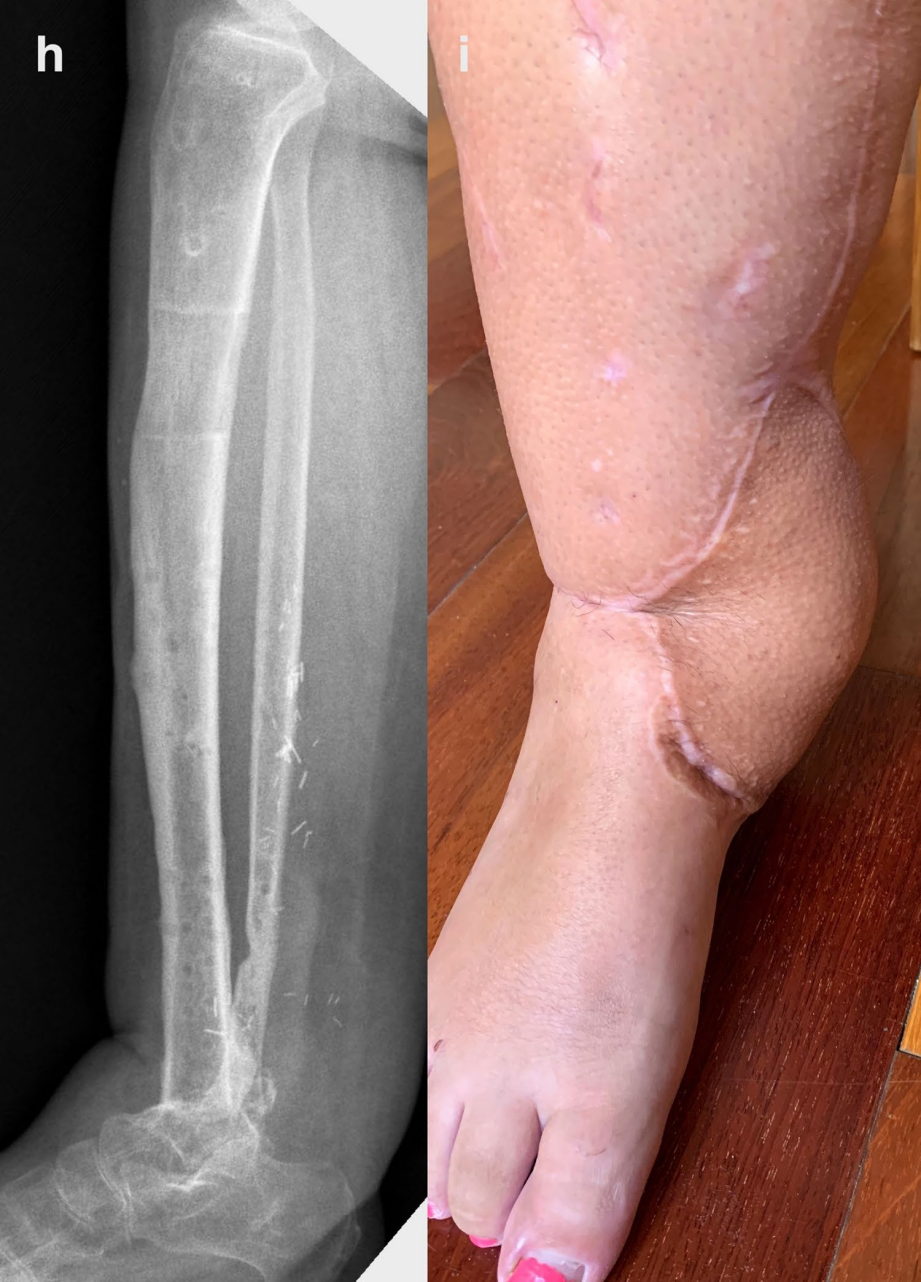
Images demonstrating the final **h** radiographic (lateral view) and **i** clinical appearance once the bone has united and the frame has been removed, with a solid tibiotalar arthrodesis

The third and final element of the three-stage protocol is the Bone Reconstruction Phase (BRP). The objective of delaying this last stage is to reconstruct the bone defect only after the infection is fully controlled and coverage has been secured. During this stage the temporary external fixator is removed, the flap is lifted, and the PMMA spacer extracted (Fig. 2e). Repeat debridement is performed and specimens obtained to confirm infection eradication. DO [16] based procedures are our technique of choice for reconstruction of massive segmental tibial defects in adults. For small defects (< 1 cm) we simply shorten the leg through monofocal compression, with or without autologous cancellous bone graft. For defects smaller than 4 cm, the shortening-lengthening procedure is used; for defects larger than 4 cm, bone transport techniques [17] are preferred. The selected external device is applied in a standard fashion (Fig. 2f, g). The osteotomy is most often performed percutaneously using a Gigli saw [18]. Distraction begins 10–14 days after the third-stage procedure, initially at 1 mm/day in four equal increments, with the rate later adjusted according to regenerate bone formation. The docking site was not routinely modified after completing transport; once consolidation was achieved, the fixator was removed (Fig. 3h, i), and full weight-bearing begun with a functional boot for 6 weeks. After external frame removal, patients were brought in to our outpatient department at 2 weeks, 1, 3 and 6 months, and 1 year after surgery. Beyond this period, if no complications had been identified, patients were followed on a bi-annual basis. Although the orthopedic surgeon is the team leader in the decision-making process during follow-up, both plastic surgeon and infectious diseases specialist (members of our multidisciplinary unit) are involved in the follow-up of these patients.

Demographic factors and clinical characteristics were summarized as means and percentages for categorical variables. Means were calculated for continuous variables. Fisher’s exact test was used to analyze the independency of success rate and satisfaction levels of the different groups. Statistical significance was established for *p* values < 0.05. All calculations were made using R software version 4.0.2.

## Results

From January 2010 through December 2018, a total of 31 complex infected tibial injuries with combined bone defects and soft-tissue loss were treated using this three-stage limb salvage protocol. Most patients came from other centers, where they had undergone multiple previous unsuccessful operations (average 3.4 previous operations; range 0–15) before being referred to our center. Demographic details and patient characteristics are summarized in Table 1**.**

**Table 1:** Demographic details and patient characteristics

Variables	Patients (*n* = 31)
Sex (M, males; F, females)	27 M/4 F
Age (years)	41 (18–72)
Follow-up (months)	48 (15–110)
Risk factors	
Smoking (> 5 cigarettes/day)	15 (48.4%)
DM (diabetes mellitus)	3 (9.7%)
Alcoholism (> 30 g/day)	4 (12.9%)
Obesity (BMI > 30)	2 (6.4%)
Mechanism of injury	
Traffic accident	18 (58.0%)
Fall from height	8 (25.8%)
Others	5 (16.1%)
Bone segment affected	
Proximal, AO type 41	2 (6.45%)
Midshaft, AO type 42	15 (48.4%)
Distal, AO type 43	14 (45.2%)
Type of fracture (GA classification)	
Open	22 (71.0%)
I and II	3
III	18
IIIA	5
IIIB	10
IIIC	3
Not known	1
Closed	9 (29.0%)
Prior surgeries (per patient)	3.4 (0–15)

*BMI* body mass index, *AO* AO/OTA classification system, *GA* Gustilo-Anderson classification

During the first stage, the most frequently isolated microorganisms were *Staphylococcus aureus* and *Enterobacter cloacae* (9.7% each, 3/31). Polymicrobial infection was detected in 11 patients (35.5%). In eight patients (25.8%), cultures tested negative despite clearly exhibiting criteria indicative of infection.

The mean soft-tissue defect was 124 cm^2^ (6–600 cm^2^). NPWT was used in 90.3% (28/31) of the cases between the first and second surgical stages. All patients required flap coverage, which was carried out in an average of 6.5 days (2–23) after the first stage. A free flap was performed in 67.7% (21/31) of the cases in this series, with the anterolateral thigh (ALT) flap most frequently used (54.8%). The mean bone defect measured 5.4 cm (1–23 cm). Fifteen patients (51.7%) underwent bone transport, where the mean bone defect was 8 cm (4–23 cm). Eleven patients (37.9%) had an acute-shortening procedure without bone lengthening; the mean defect was 1.4 cm (1–3 cm). Three patients (10.3%) underwent a shortening-lengthening procedure, with a mean bone defect of 3 cm (range 2–4 cm). Treatment characteristics and reconstructive techniques are summarized in Table 2.

**Table 2:** Treatment characteristics for complex infected tibial fractures

Variables	Patients (*n* = 31)
Bone defect size (cm)	5.4 (1–23)
Soft tissue defect (cm^2^)	124 (6–600)
Microbiological cultures (1st surgical stage)	
Mixed Flora	11 (35.5%)
Negative	8 (25.8%)
*Staphylococcus aureus*	3 (9.7%)
*Enterobacter cloacae*	3 (9.7%)
Other*	6 (19.4%)
Bone reconstruction technique (*n* = 29)	
Bone transport	15 (51.7%)
Acute shortening	11 (37.9%)
Shortening/lengthening	3 (10.3%)
External fixation (*n* = 29)	
Monolateral (LRS)	12 (41.4%)
Hexapod (TL Hex)	11 (37.9%)
Ilizarov (TL/Clickit-CF)	6 (20.7%)
Soft tissue coverage	
Free fasciocutaneous flap	17 (54.8%)
Anterolateral thigh	17
Local fasciocutaneous flap	3 (9.7%)
Keystone Perforator Island	3
Free muscle flap	4 (12.9%)
Gracilis	4
Local muscle flap	7 (22.6%)
Medial Gastrocnemius	4
Medial Gastrocnemius + Hemisoleus	1
Hemisoleus	1
Peroneus Brevis	1

*LRS* limb reconstruction system (Orthofix, Sommacampagna, Verona, Italy), *TL-HEX* Truelok Hexapod (Sommacampagna, Verona, Italy); Clickit-CF (S Mikai S.p.A, Genova, Italy), *TL* Truelok (Sommacampagna, Verona, Italy)

*Other—isolated microorganisms: *Acinetobacter baumanii, Cutibacterium acnes, Pseudomona* spp., SPCN (no epidermidis),* Streptococcus* spp.,* Candida parapsilosis*

After the mean follow-up of 48 months, limb salvage was achieved in 29 of 31 patients (93.5%). Two patients underwent a delayed amputation after initial treatment failure; one was due to the inability to eradicate infection. The union rate in successfully salvaged limbs was 100%. At final follow-up, no signs of clinical infection were observed among the salvaged limbs.

The average treatment time (first surgical stage to external fixator removal) was 45 weeks (range 12–107). The external fixation time (EFT) in the bone transport group was 56 weeks. The EFT in the shortening-lengthening group was 41 weeks, while in the acute shortening group it was 34 weeks. The average bone healing index (BHI) in the bone transport group was 1.6 months/cm of bone reconstruction. The average BHI in the shortening-lengthening group was 2.2 months/cm.

Complications encountered are summarized in Table 3. In nine cases of bone transport (9/15) and in one case of shortening-lengthening (1/3), a non-union at the docking site was observed. In those cases, the docking site was debrided and an iliac crest autologous bone graft was used to achieve union. There were five cases of flap failure (16.7%, 5/31); the failure rate of local flaps was 30% (3/10), while for free flaps it was 9.5% (2/21). However, this difference was not statistically significant (*p* = 0.2955).

**Table 3:** Complications and inherent interventions related to reconstruction of infected tibial fractures

Variables	Patients (*n* = 31)	Interventions
Complications (per patient)	1.1	
Unexpected interventions	25	
Docking site nonunion (*n* = 18*)	11 (61.1%)	
Bone transport	10/15	Debridement + auto-cancellous bone graft from iliac crest
Shortening/lengthening	1/3	Debridement + auto-cancellous bone graft from iliac crest
Pin track infections	10 (32.2%)	
Local	7	Local care + systemic antibiotic
Loosening pin	3	Exchange of a pin
Persistent infection	1	Amputation
Flap failures (*n* = 30)	5 (16.7%)	
Local flap	3 (30%)	
Keystone perforator Island	2	Dorsalis pedis, Dorsalis pedis
Peroneus brevis	1	ALT
Free flap	2 (9.5%)	
ALT (anterolateral thigh)	2	ALT, Amputation
External fixator realignment	5	Surgery
Varus deformity of the ankle	1	Percutaneous medial plate
Late fracture of distal tibia	1	Percutaneous monolateral fixator without approaching fracture site

*By definition, docking site nonunion can only occur in “Bone Transport technique” and “Shortening/Lengthening technique”

Functional outcomes were analyzed in the 29 patients with successful limb salvage; the details are summarized in Table 4. The mean VAS score was 1.0 (range 0–5), and 69% of the patients (20/29) experienced no pain after the procedure. The mean LEFS score was 57.5 (range 43–74), indicating that most patients were able to walk more than 1 km or go up or down ten stairs. The ASAMI bone score was excellent/good in 100% of the cases; the ASAMI functional score was excellent in 58.6%, good in 27.6%, and poor in 13.8%. All of the poor results were associated with an inability to return to work, although 83% of those who were of working age were able to return to their previous work activities (Table 4).

**Table 4:** Patient-reported functional outcomes and self-administered satisfaction

Variables	Patients (*n* = 29*)
Pain (VAS)	1 (0–5)
Return to work (RTW)	
Yes	20 (69%)
No	4 (13.8%)
Already retired before the lesion	5 (17.2%)
RTW among non-retired patients	20/24 (83.3%)
Walking aid	
None	24 (82.7%)
Crutch or cane	5 (17.3%)
Limb length discrepancy (cm)	1.4 (0–3.8)
LEFS	57.5 (43–74)
ASAMI Bone Score	
Excellent	21 (72.4%)
Good	8 (27.6%)
Fair	–
Poor	–
ASAMI Functional Score	Excellent
Excellent	17 (58.6%)
Good	8 (27.6%)
Fair	–
Poor	4 (13.8%)
SAPS	
Very satisfied	85.7%
Moderately satisfied	14.3%
Pain	
Very satisfied	89.3%
Moderately satisfied	10.7%
Do home/yard work	
Very satisfied	71.4%
Moderately satisfied	28.6%
Recreational activities	
Very satisfied	50%
Moderately satisfied	42.9%
Moderately dissatisfied	7.1%

*LEFS* The Lower Extremity Functional Scale, *ASAMI* Association for the Study and Application of the Method of Ilizarov, *SAPS* the self-administered patient satisfaction survey

*One of the patients could not take part in the SAPS and LEFS evaluations due to death from lung cancer during the follow-up period; however the rest of this patient’s data was collected before the patient succumbed to the disease

## Discussion

In this series of patients with chronic fracture-related infection (FRI) and combined bone and soft-tissue defects, managed with such a comprehensive three-stage limb-salvage protocol, we demonstrate a limb-salvage rate of 93.5% (29 out of 31 patients) after a median follow-up of 48 months. Employing our three-stage strategy, including formal soft-tissue reconstruction, we found no infection relapse among the patients with successful limb reconstructions. Functional and patient-satisfaction outcomes were encouraging, showing that our limb-salvage protocol is a valid therapeutic option in such limb-threatening situations and confirming the study’s initial hypothesis.

Two-stage reconstruction for infected post-traumatic injuries has been widely accepted for the past 30 years, with reported success rates of 89–94% [5, 6]. Others have reported favorable results with single-stage protocols [7–9]. Mifsud et al. [9] recently presented 57 cases of chronic osteomyelitis (COM) and infected non-union resolved in a single reconstruction with simultaneous debridement, Ilizarov method and free muscle flap transfer, with high rates of infection eradication (96.5%) and bone union (91.2%). A 5.3% rate of flap failure was observed. Spiegl et al. [19] presented a prospective series of 25 patients treated with multi-stage protocol with consolidation of 76% and a major complication rate of 0.52 per patient. To the best of our knowledge, there are few studies describing clinical features, treatment protocols and outcomes in such a difficult-to-treat scenario of limb threatening infected (LTI) tibial injuries [9, 19, 20].

The fundamental principle governing any infected-limb-salvage strategy is complete debridement of all necrotic and infected tissues, together with dead-space management, skeletal stabilization, and targeted antimicrobial treatment [5, 6]. Once the initial infection is controlled (iLDC), soft-tissue reconstruction often determines whether a limb can be successfully salvaged. Temporary NPWT between the first and second stage is used to manage such open wounds. It isolates the wound from the hospital environment, preventing recolonization and nosocomial contamination until the following surgery, which should be completed within a maximum of 10 days. Although with acute open fractures the value of NPWT has been debated [21] for its potential for colonization, during the second stage a new radical debridement, sample-taking and spacer change are performed to avoid new colonization.

The average soft-tissue defect in our cases was 124 cm^2^, with an overall flap failure rate of 16.7%. Local flap failure rate was 30%, with 9.5% for free flaps. Although not a significant difference, probably reflecting the small sample size, these results reinforce our conviction that a free tissue transfer is the best choice in this scenario. Local flap options are limited, particularly for the middle and distal segments of the tibia, with reports demonstrating better success rates with free flaps [7]. Several studies have demonstrated similar success rates between muscular and fasciocutaneous flaps, in terms of flap survival, complications, and functional recovery in the lower extremity [22]. The main advantage of fasciocutaneous flaps is their ease of subsequent elevation, making them ideal for staged protocols like ours; fasciocutaneous free flaps are therefore preferred, particularly the ALT. Although, free flaps are challenging due to their inherent complexity; microvascular reconstruction can be difficult due to vascular thrombosis, perivascular fibrosis or previous vessel lesion during the initial injury, with the appropriate microsurgical expertise free flaps are much safer [23]. Donor site morbidity and the simultaneous use of external fixator can also present challenges.

Bone reconstruction of infected tibial non-unions with segmental bone defects is a formidable challenge. Despite the encouraging evolution of reconstruction techniques, there is no consensus as to which is the ideal procedure. The current *biological* techniques for reconstruction of massive bone defects are limited; they can be divided into two main groups: bone-replacement techniques (autologous cancellous bone grafting, induced membrane technique, or free vascularized bone grafting [24–26] and bone-regeneration techniques (techniques based on distraction osteogenesis).

Wen et al*.* [27] recently published a retrospective study comparing DO, free vascularized fibular transfer, and the Masquelet technique in treatment of 371 post-traumatic long-bone defects, and observed no differences between methods with respect to complication rates, long-term quality of life, chronic pain, or ambulatory status. It should be noted that in the aforementioned study the authors included injuries to the tibia and femur, only 21% of the injuries were infected, and only 10% of the cases required soft-tissue reconstruction—a global scenario very different from ours. In our unit, DO-based techniques are preferred, as they provide unique advantages for infected segmental bone defects of the lower limb. However, the most appropriate bone reconstruction technique is carefully selected for each patient. Generally, bone transport is most suitable for defects > 3.5–4 cm, shortening-lengthening for defects from 1 to 3.5 cm, and acute shortening for defects < 1 cm. Using this protocol resulted in a bone union rate of 100%, with 100% of ASAMI bone scores good/excellent. The benefits of external fixation and DO techniques in the presence of infection are well known, including use of temporary implants far from the infected area, preservation of local vascularity, intraoperative flexibility, and the ability to successfully reconstruct very large bone defects without the restrictions of autograft availability or donor site morbidity. Most importantly, the characteristics of the regenerate bone are most similar to that which was lost [28].

In this study, the most obvious disadvantage of external fixation was the prolonged course, with an average treatment time of 45 weeks. For bone transport our EFT was 56 weeks, which is comparable to other reports. Wang et al*.* [29] reported EFT at 48 weeks in 15 infected tibial non-unions treated with bone transport using a circular frame, while Hohmann et al*.* [30] reported EFT of 42 weeks in 32 infected and aseptic tibial non-unions treated with bone transport. In a recent meta-analysis, Aktuglu et al*.* [27] evaluated Ilizarov methods for the treatment of infected or non-infected critical-size tibial bone defects, finding a mean EFT of 10.7 months (range 2.5–23.2).

In the authors’ opinion, these are complex techniques that are best performed in experienced bone and joint infection centers which can provide a multidisciplinary team. However, even in specialized centers the number of complications is not trivial, with an average of 1.1 per patient in this cohort, comparable to other studies [26]. Pin-site infection is the most common complication of external fixation [31, 32]; we experienced 32.2% symptomatic pin-site infections, but only three of these required wire/pin replacement. Interestingly, there were 61.1% non-unions at the docking site requiring debridement and autogenous cancellous bone grafting, all resulting in union. The ideal docking-site management protocol is not well established. Ilizarov, classically, proposed a simple compression of the bone ends (closed docking site) with eventual periods of distraction. Some authors, on the other hand, suggest systematically approaching the bone ends (open docking site) with bone grafting as an additional procedure [33]. Based on the results of this study and other previous investigations [16], currently the docking site is systematically approached for debridement and an iliac crest graft in all cases of bone transport > 4 cm.

Psychological impact and the likelihood of restoration of function are important considerations, as results can be disappointing when expectations are high [34] and the final outcome is suboptimal. Pain is reported to persist in over 50% of limb salvage patients [35], but in our series the mean VAS pain rating was 1.0, and 69% of the patients experienced no pain after completing the reconstruction. The ASAMI functional score was good-to-excellent in 86%. Moreover, all poor functional results were related to the incapacity to return to work, considered a reliable measure of treatment outcome [36]. In fact, 83% of our patients who were of working age were able to return to their previous work activities; surprisingly high when compared to some prior reports [4].

We recognize both the strengths and limitations of the present research. The first limitation lies in the study’s retrospective nature. Retrospective studies rely on chart notes from which important data may be lacking, increasing bias incidence. A second major limitation is our lack of a comparison group; absence of a control group makes it impossible to compare results directly with other limb-salvage protocols in the same scenario. Our third limitation concerns sample size. Our patient cohort was small, although it was comparable in size to other studies with similar patients; this limited the study’s statistical power, and therefore the generalizability of its results. Fourth, the inherent heterogeneity of the study cohort resulted in a broad range of injuries, which rendered them difficult to analyze and compare objectively. Finally, we recognize that all care was provided at a single, high-volume, specialized center; it is difficult to extrapolate these results to less-experienced units. The consistencies of our well-established protocol and strict follow-up add, in our opinion, to the homogeneity and validity of our study. Studies employing prospective data retrieval, larger patient bases and more extensive follow-up are undoubtedly needed.

## Conclusions

The proposed three-stage surgical approach for the management of complex infected tibial injuries, with combined bone and soft-tissue defects, yields high rates of both infection eradication and successful limb salvage. This protocol consistently delivers good functional outcomes and high levels of self-reported patient satisfaction, despite its demand for more resources than a one-step approach, and an inherent potential for complications.

## Abbreviations

DO: Distraction osteogenesis
VAS: Visual Analogue Scale
RTW: Return to work
SAPS: Self-Administered Patient Satisfaction Scale
ASAMI: Association for the Study and Application of the Methods of Ilizarov classification
LEFS: Lower-Extremity Functional Scale
TSP: Three-stage surgical treatment protocol
iLDC: Labelled infected-limb damage control
PMMA: Polymethylmethacrylate
NPWT: Negative pressure wound therapy
WRP: Wound reconstruction phase
BRP: Bone reconstruction phase
ALT: Anterolateral thigh flap
EFT: External fixation time
BHI: Bone Healing Index
FRI: Fracture related infection
COM: Chronic osteomyelitis
LTI: Limb-threatening injuries

## References

[1] Larsen P, Elsoe R, Hansen SH (2015). Incidence and epidemiology of tibial shaft fractures. Injury.

[2] Audigé L, Griffin D, Bhandari M (2005). Path analysis of factors for delayed healing and nonunion in 416 operatively treated tibial shaft fractures. Clin Orthop.

[3] Bosse MJ, Teague D, Reider L (2017). Outcomes after severe distal tibia, ankle, and/or foot trauma: comparison of limb salvage versus transtibial amputation (OUTLET). J Orthop Trauma.

[4] Busse JW, Jacobs CL, Swiontkowski MF (2007). Complex limb salvage or early amputation for severe lower-limb injury: a meta-analysis of observational studies. J Orthop Trauma.

[5] McNally M, Nagarajah K (2010). Osteomyelitis. Orthop Trauma.

[6] Heitmann C, Patzakis MJ, Tetsworth KD (2003). Musculoskeletal sepsis: principles of treatment. AAOS Instr Course Lect Ser.

[7] Chan JKK, Ferguson JY, Scarborough M (2019). Management of post-traumatic osteomyelitis in the lower limb: current state of the art. Indian J Plast Surg.

[8] McNally M, Ferguson J, Kugan R (2017). Ilizarov treatment protocols in the management of infected nonunion of the Tibia. J Orthop Trauma.

[9] Mifsud M, Ferguson Y, Stubbs DA (2020). Simultaneous debridement, Ilizarov reconstruction and free muscle flaps in the management of complex tibial infection. JBJI.

[10] Obremskey WT, Metsemakers WJ, Schlatterer DR (2020). Musculoskeletal infection in orthopaedic trauma: assessment of the 2018 international consensus meeting on musculoskeletal infection. J Bone Jt Surg Am.

[11] Morgenstern M, Kühl R, Eckardt H (2018). Diagnostic challenges and future perspectives in fracture-related infection. Injury.

[12] Mahomed N, Gandhi R, Daltroy L, Katz JN (2011). The Self-Administered Patient Satisfaction Scale for primary hip and knee arthroplasty. Arthritis.

[13] Paley D, Catagni MA, Argnani F (1989). Ilizarov treatment of tibial nonunions with bone loss. Clin Orthop Relat Res.

[14] Cruz-Díaz D, Lomas-Vega R, Osuna-Pérez MC (2014). The Spanish lower extremity functional scale: a reliable, valid and responsive questionnaire to assess musculoskeletal disorders in the lower extremity. Disabil Rehabil.

[15] Stravinskas M, Horstmann P, Ferguson J (2016). Pharmacokinetics of gentamicin eluted from a regenerating bone graft substitute: in vitro and clinical release studies. Bone Jt Res.

[16] Corona PS, Ramirez-Nuñez LJ, Amat C (2017). Outcome of oscillating saw open osteotomy in two-stage lower extremity bone transport with monolateral frame. Injury.

[17] Tetsworth K, Paley D, Sen C (2017). Bone transport versus acute shortening for the management of infected tibial non-unions with bone defects. Injury.

[18] Paktiss AS, Gross RH (1993). Afghan percutaneous osteotomy. J Pediatr Orthop.

[19] Spiegl U, Pätzold R, Friederichs J (2013). Clinical course, complication rate and outcome of segmental resection and distraction osteogenesis after chronic tibial osteitis. Injury.

[20] Rein S, Hörnig J, Houschyar KS (2020). Microsurgical soft tissue reconstruction in lower extremity osteitis. Handchir Mikrochir Plast Chir.

[21] Costa ML, Achten J, Bruce J, WOLFF Trial 2018 (2018). Effect of negative pressure wound therapy vs standard wound management on 12-Month disability among adults with severe open fracture of the lower limb: The WOLLF randomized clinical trial. JAMA.

[22] Lee Z-H, Abdou SA, Daar DA (2019). Comparing outcomes for fasciocutaneous versus muscle flaps in foot and ankle free flap reconstruction. J Reconstr Microsurg.

[23] Xiong L, Gazyakan E, Kremer T (2016). Free flaps for reconstruction of soft tissue defects in lower extremity: a meta-analysis on microsurgical outcome and safety. Microsurgery.

[24] Capanna R, Bufalini C, Campanacci M (1993). A new technique for reconstructions of large metadiaphyseal bone defects. Orthop Traumatol.

[25] Capanna R, Campanacci DA, Belot N (2007). A new reconstructive technique for intercalary defects of long bones: the association of massive allograft with vascularized fibular autograft. Long-terms results and comparison with alternative techniques. Orthop Clin N Am.

[26] Moghaddam A, Ermisch C, Fischer C (2017). Tibial defects and infected non-unions: treatment results after Masquelet technique. Orthopäde.

[27] Wen G, Zhou R, Wang Y (2019). Management of post-traumatic long bone defects: a comparative study based on long-term results. Injury.

[28] Aktuglu K, Erol K, Vahabi A (2019). Ilizarov bone transport and treatment of critical-sized tibial bone defects: a narrative review. J Orthop Traumatol.

[29] Wang H, Wei X, Liu P (2017). Quality of life and complications at the different stages of bone transport for treatment infected nonunion of the tibia. Medicine (Baltimore).

[30] Hohmann E, Birkholtz F, Glatt V (2017). The “Road to Union” protocol for the reconstruction of isolated complex high-energy tibial trauma. Injury.

[31] Tetsworth K, Dlaska C (2015). The art of tibial bone transport using the Ilizarov fixator. Tech Orthop.

[32] Kazmers NH, Fragomen AT, Rozbruch SR (2016). Prevention of pin site infection in external fixation: a review of the literature. Strateg Trauma Limb Reconstr.

[33] Lovisetti G, Sala F (2013). Clinical strategies at the docking site of distraction osteogenesis: are open procedures superior to the simple compression of Ilizarov?. Injury.

[34] Akula M, Gella S, Shaw CJ (2011). A meta-analysis of amputation versus limb salvage in mangled lower limb injuries—the patient perspective. Injury.

[35] Tay W-H, de Steiger R, Richardson M (2014). Health outcomes of delayed union and nonunion of femoral and tibial shaft fractures. Injury.

[36] MacKenzie EJ, Morris JA, Jurkovich GJ (1998). Return to work following injury: the role of economic, social, and job-related factors. Am J Public Health.

